# An In Vitro Comparison of the Compression Resistance and Tear Strength of Interocclusal Recording Materials After Immersion in a Chemical Disinfectant

**DOI:** 10.7759/cureus.104370

**Published:** 2026-02-27

**Authors:** HimaBindu G, Jhansi Muram, Raja R N, Tejeswar R B, Shreya Bukkapatnam, Sonu B

**Affiliations:** 1 Department of Prosthodontics, Chadalawada Krishna Srinivasa (CKS) Teja Institute of Dental Sciences and Researc, Tirupathi, IND; 2 Department of Prosthodontics, Chadalawada Krishna Srinivasa (CKS) Teja Institute of Dental Sciences and Researc, Tirupati, IND; 3 Department of Prosthodontics, Pulla Reddy G Dental College and Hospital, Kurnool, IND; 4 Department of Prosthodontics, Rajarajeswari Dental College and Hospital, Bangalore, IND; 5 Department of Dentristry, National Medical Center Specialty Hospital, Abudhabi, ARE

**Keywords:** aluwax, compression resistance, disinfection, interocclusal recording materials, occlusal registration, polyvinyl siloxane, tear strength, zinc oxide eugenol

## Abstract

Aim

The purpose of the study is to evaluate the compression resistance and tear strength of interocclusal recording materials after immersion in a chemical disinfectant.

Methods

A total of 120 specimens were prepared, with 30 samples for each material type. These were further divided into two subgroups: a control group (no disinfection) and an experimental group (subjected to disinfection), with 15 specimens in each experimental group. The specimens in the experimental group were immersed in a commonly used chemical disinfectant, such as 2% glutaraldehyde for 30 min, while the control group was stored in a dry environment under ideal conditions without exposure to any disinfectant. The specimens were made into cylindrical and dumbbell shapes for compressive and tear strength testing, respectively, using a universal testing machine, and the values obtained were subjected to statistical analysis.

Results

The findings were analysed by ANOVA and Tukey's post hoc test in SPSS version 27.0 (IBM Inc., Armonk, New York). The results revealed that polyvinyl siloxane (PVS) exhibited the highest compression resistance and tear strength, both before and after disinfection, demonstrating superior mechanical stability. Aluwax showed moderate mechanical performance, with slight changes after immersion. In contrast, zinc oxide eugenol (ZOE) exhibited the lowest resistance, showing significant degradation in both properties post-immersion. Statistical analysis confirmed a significant impact of disinfection on material performance (p<0.05), particularly for ZOE.

Conclusion

Among the tested materials, PVS demonstrated the best overall performance, maintaining its mechanical integrity post-disinfection, making it the preferred choice for clinical applications requiring durability and accuracy. Aluwax retained moderate properties, while ZOE was the least reliable due to its significant degradation after immersion. These findings emphasize the need for careful material selection in prosthodontics to ensure clinical reliability while maintaining effective infection control.

## Introduction

Accurate recording of centric relation is fundamental to successful prosthetic diagnosis and treatment planning. Interocclusal records are essential for representing an unstrained maxillomandibular relationship and for mounting casts on an articulator [1]. The primary requirements for an ideal centric relation record include accurate horizontal mandibular positioning, uniform vertical pressure, and dimensional stability until cast mounting [2].

Several techniques exist for recording maxillomandibular relations, including graphic, functional, and cephalometric methods; however, direct interocclusal records are most commonly used due to their simplicity and clinical practicality [3]. Errors in occlusion associated with these records may result from patient-related factors, clinician-induced inaccuracies, and the physical properties of the recording materials, as well as laboratory handling [4].

Interocclusal recording materials, such as impression plaster, dental waxes, zinc oxide eugenol paste, acrylic resin, hydrocolloids, polyether, and polyvinyl siloxane, are modified impression materials designed for improved handling and accuracy. These materials must maintain adequate dimensional stability, tear strength, and compressive resistance, particularly following disinfection procedures.

With increasing awareness of infectious diseases such as hepatitis B and acquired immunodeficiency syndrome (AIDS), stringent disinfection protocols have become mandatory. Commonly used chemical disinfectants include sodium hypochlorite and glutaraldehyde, along with other agents and newer physical disinfection methods. However, interactions between disinfectants and recording materials may adversely affect their mechanical properties. Studies report minimal dimensional changes following immersion in sodium hypochlorite and glutaraldehyde, but alterations in surface characteristics and compressive resistance have been observed [5,6].

Therefore, compatibility between interocclusal recording materials and disinfection protocols is critical to ensure clinical reliability. This study aims to evaluate and compare the compression resistance and tear strength of selected interocclusal recording materials after immersion in chemical disinfectants to determine their suitability for clinical use.

## Materials and methods

Study design and ethical approval

This in vitro experimental study was conducted in the Department of Prosthodontics after obtaining institutional ethical clearance (CKS/PROSTHO/22-23/01). The study aimed to evaluate the effect of chemical disinfection on the compression resistance and tear strength of selected interocclusal recording materials. The experimental protocol was developed with reference to American National Standards Process Resources (ANSI)/American Dental Association (ADA) Specification No. 19 for dental elastomeric impression materials and relevant Advancing Standards Transforming Markets (ASTM) testing standards [7].

Sample size determination

The sample size was determined based on previously published in vitro studies evaluating the mechanical properties of dental materials [2]. Assuming a moderate effect size, a statistical power of 80%, and a significance level of 5%, a minimum of 10 specimens per group was considered sufficient to detect statistically significant differences. Accordingly, an equal number of specimens were prepared for each study group.

Materials and specimen preparation

Three interocclusal recording materials-polyvinyl siloxane, zinc oxide eugenol paste, and Aluwax-were included in the study. All materials were manipulated in strict accordance with the manufacturers’ instructions to maintain uniformity.

For tear strength evaluation, specimens were fabricated using a custom-made metal mold conforming to ASTM D624 type C specifications [8]. The mold consisted of upper and lower halves with orientation guides to ensure accurate repositioning and incorporated a centrally located V-shaped tear guide with a depth of 2 mm. For compression resistance testing, standardized cylindrical stainless steel molds with an internal diameter of 18 mm and a height of 20 mm were used. The mixed materials were carefully packed into the molds to avoid air entrapment. After complete setting, the specimens were retrieved and visually inspected, and any specimen with surface defects or irregularities was excluded from testing.

All materials were manipulated in strict accordance with the manufacturers' instructions to maintain uniformity. To minimize operator-related variability, all materials were manipulated, and all specimens were fabricated by a single trained operator following standardized protocols. This approach was adopted to reduce inter-operator variability and enhance consistency across the study groups.

Disinfection protocol and group allocation

All specimens except the control group were immersed in 2% glutaraldehyde solution for 30 minutes in accordance with established disinfection protocols [9]. Following immersion, the specimens were thoroughly rinsed under running distilled water for 30 seconds to remove residual disinfectant and were subsequently air-dried at room temperature prior to mechanical testing. The specimens were categorized into four groups: group A (control, no disinfection), group B (polyvinyl siloxane after disinfection), group C (zinc oxide eugenol after disinfection), and group D (Alu-wax after disinfection).

Mechanical testing

Compression resistance was measured using a universal testing machine. The specimens were tested at a crosshead speed of 1 mm/min. Each specimen was subjected to a compressive load of 25 N, and the maximum load sustained was recorded. Compressive strength (σ) was calculated in megapascals (MPa) using the formula: σ = F/A, where F represents the maximum applied load, and A denotes the cross-sectional area of the specimen in mm².

Tear strength was assessed by subjecting dumbbell-shaped specimens to tensile loading in the universal testing machine until rupture. Tear strength testing was conducted at a crosshead speed of 500 mm/min in accordance with ASTM D624 specifications. Tear strength (T) was calculated in N/mm using the formula: T = F/D, where F is the tearing force at failure, and D represents the specimen thickness.

Statistical analysis

Statistical analysis was performed using appropriate statistical software. Mean and standard deviation were calculated for all groups. Intergroup comparisons were carried out using one-way analysis of variance (ANOVA), followed by Tukey's post hoc test for pairwise comparisons. A p-value of ≤0.05 was considered statistically significant.

## Results

**Figure 1:**
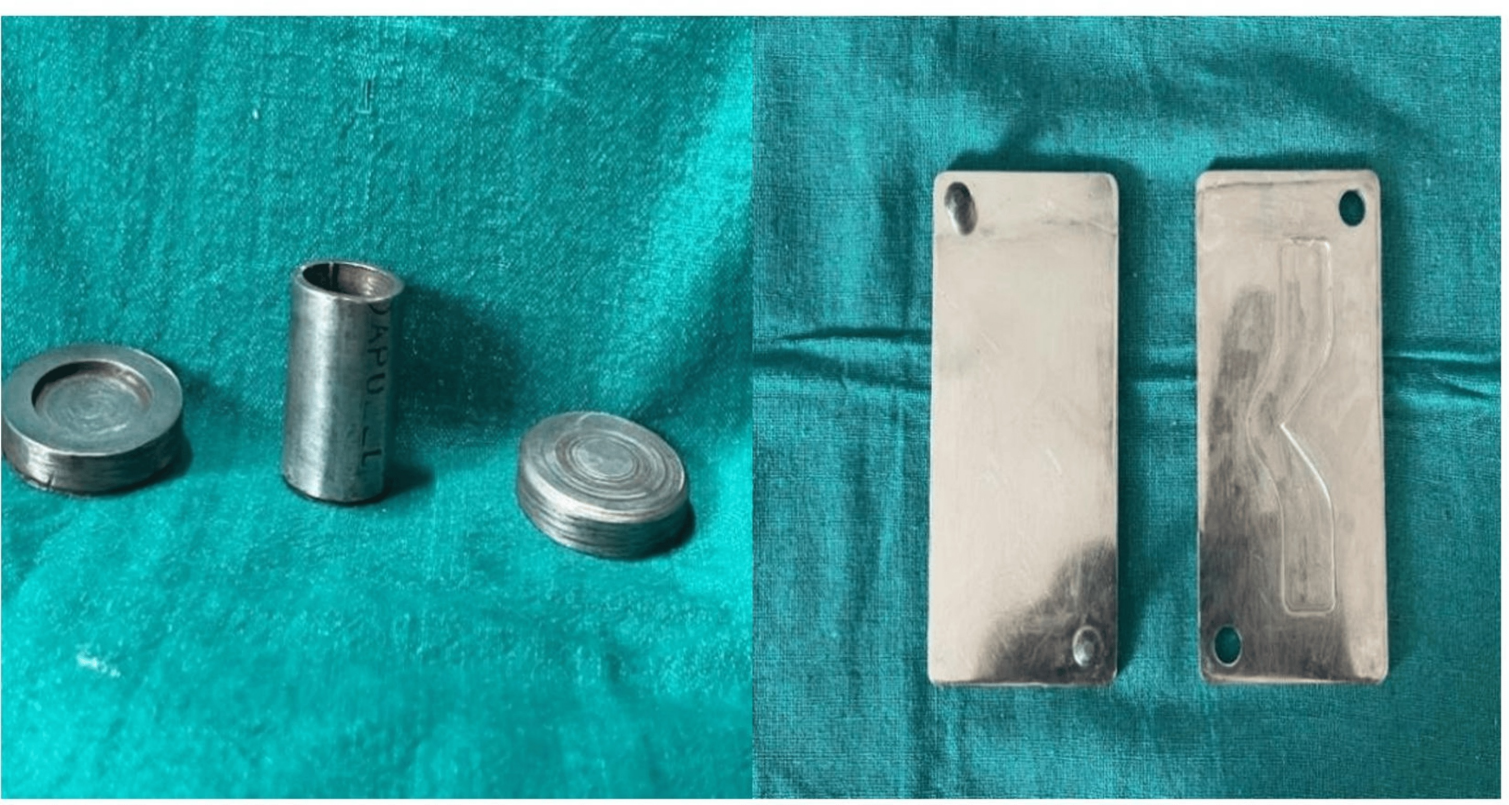
Metal molds

**Figure 2:**
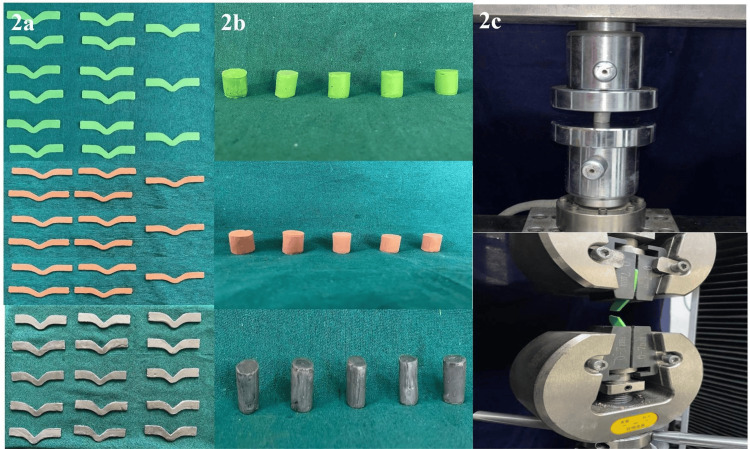
a,b) Samples of PVS, ZOE, Aluwax; c) Test samples placed in between the jaws of the universal resting machine PVS - polyvinyl siloxane; ZOE - zinc oxide eugenol

A total of specimens fabricated from three interocclusal recording materials, polyvinyl siloxane (PVS), zinc oxide eugenol (ZOE), and Aluwax, were evaluated to determine compression resistance and tear strength before and after immersion in a 2% glutaraldehyde disinfectant. The experimental setup and specimen preparation are illustrated in Figure 1 and Figure 2. Statistical analysis was performed using descriptive measures and inferential testing.

Compression resistance

Before immersion, a one-way analysis of variance demonstrated a statistically significant difference in compression resistance among the tested materials (p<0.001). PVS exhibited the highest mean compression resistance, followed by Aluwax and ZOE. After immersion, significant differences among the groups persisted (p<0.001), with PVS maintaining the greatest resistance and ZOE showing the lowest values.

Comparison of pre- and post-immersion compression resistance within each material using independent samples t-tests revealed statistically significant changes for all groups. PVS and Aluwax demonstrated increased compression resistance after immersion, whereas ZOE showed a reduction. These findings indicate that chemical disinfection significantly influences the compressive behavior of interocclusal recording materials (Table 1).

**Table 1:** Compression resistance of interocclusal recording materials before and after immersion p-values were obtained using an independent samples t-test comparing compression resistance before and after immersion PVS - polyvinyl siloxane; ZOE - zinc oxide eugenol

Material	Mean ± SD (without immersion)	Mean ± SD (after immersion)	Test statistic (t)	p-value
ZOE	1.14 ± 0.35	0.50 ± 0.33	3.60	0.010
Aluwax	2.16 ± 0.65	6.33 ± 2.20	6.54	<0.001
PVS	11.96 ± 2.52	15.55 ± 2.70	2.71	0.029

Tear strength

Tear strength measurements also showed statistically significant differences among the three materials prior to immersion (p<0.001), with PVS demonstrating the highest tear resistance, followed by Aluwax and ZOE. A similar pattern was observed after immersion, and intergroup differences remained statistically significant (p<0.001). Intra-group comparison indicated that immersion produced a significant increase in tear strength for PVS, while ZOE exhibited a significant decrease. No statistically significant change was observed for Aluwax. Overall, PVS consistently demonstrated superior tear strength under both conditions, suggesting enhanced durability following disinfection (Table 2).

**Table 2:** Tear strength of interocclusal recording materials before and after immersion p-values were obtained using an independent samples t-test comparing tear strength before and after immersion. PVS - polyvinyl siloxane; ZOE - zinc oxide eugenol

Material	Mean ± SD (without immersion)	Mean ± SD (after immersion)	Test statistic (t)	p-value
PVS	12.30 ± 1.50	16.85 ± 2.29	5.18	<0.001
ZOE	3.37 ± 1.34	1.59 ± 0.41	2.83	0.041
Aluwax	7.78 ± 0.99	7.64 ± 2.53	0.17	0.863

## Discussion

Successful occlusal rehabilitation aims to restore function, comfort, health, and esthetics and is critically dependent on accurate recording of maxillomandibular relations. Interocclusal registration materials play a vital role in transferring this relationship for the fabrication of fixed and removable prostheses. The accuracy and validity of centric relation records are strongly influenced by the mechanical properties of the materials used [10].

For stable articulation of maxillary and mandibular casts, both horizontal stability and a tripod of vertical support are essential. While adequate intercuspation may allow direct articulation, most prosthodontic patients present with compromised occlusion, necessitating the use of interocclusal recording materials. These materials should allow minimal resistance before setting, remain dimensionally stable after setting, accurately reproduce occlusal surfaces, and possess sufficient compressive and tear resistance.

Various interocclusal materials have evolved over time. Impression plaster provides accuracy and rigidity but is technique-sensitive and brittle [11]. Thermoplastic waxes, although economical and easy to manipulate, are prone to distortion and dimensional instability, especially following removal from the oral cavity. Aluwax, containing metallic fillers, demonstrates improved handling but remains susceptible to deformation. Zinc oxide eugenol (ZOE) paste is widely used due to its flow prior to setting and rigidity after setting; however, its brittleness, prolonged setting time, and susceptibility to fracture limit its durability [12].

Elastomeric materials, particularly addition silicone (polyvinyl siloxane), have gained popularity due to superior dimensional stability, elastic recovery, and minimal resistance to closure [13]. Despite their advantages, their mechanical behavior following disinfection remains a critical consideration.

Disinfection of interocclusal records is mandatory to prevent cross-contamination, as recommended by the CDC and ADA [13]. Chemical disinfectants such as glutaraldehyde and sodium hypochlorite are widely used; however, exposure to these agents may alter the mechanical properties of recording materials. Previous studies have reported changes in dimensional stability, surface integrity, compression resistance, and tear strength following disinfection [14].

Compression resistance reflects a material’s ability to withstand occlusal forces without deformation, while tear strength indicates resistance to fracture during removal or articulation [15]. In the present study, polyvinyl siloxane demonstrated superior compression resistance and tear strength both before and after immersion, followed by Aluwax, while ZOE consistently showed the lowest values. These findings indicate that PVS maintains mechanical integrity even after disinfection, whereas ZOE is significantly compromised.

The results of this study are consistent with earlier investigations reporting superior performance of PVS due to its cross-linked polymer structure and elastic recovery [16-18]. Studies by Madaan et al. and others also reported a significant reduction in the mechanical properties of ZOE after immersion, supporting the present findings [19]. Tear strength values observed for PVS, Aluwax, and ZOE in this study align with previous reports by Hariprasad et al., Lawson et al., and others [20, 21].

Several studies have further confirmed that PVS remains dimensionally stable and mechanically resilient following chemical disinfection, whereas ZOE exhibits surface degradation and brittleness [8, 22]. However, conflicting reports suggest that prolonged immersion or variation in disinfectant type may affect elastomeric materials, highlighting the importance of controlled disinfection protocols [3, 23, 24].

Overall, this study reinforces that polyvinyl siloxane is the most reliable interocclusal recording material with respect to compression resistance and tear strength following disinfection. Aluwax demonstrates moderate stability, while zinc oxide eugenol shows significant deterioration. These findings emphasize the need for careful material selection to ensure accuracy, durability, and clinical success under mandatory infection control protocols.

Future research should incorporate in vivo designs to better simulate clinical conditions. Evaluating a wider range of disinfectants and prolonged or repeated disinfection cycles would provide deeper insight into material durability. Assessment of additional mechanical and physical properties, along with comparisons across different manufacturers and formulations, is recommended. Studies comparing conventional materials with digital bite registration systems, as well as investigations using larger sample sizes and advanced analytical methods such as finite element analysis and micro-CT, may further enhance clinical relevance.

This in vitro study may not fully replicate clinical oral conditions, including salivary exposure, temperature variations, and masticatory forces. Only a single chemical disinfectant and a short immersion duration were evaluated, which may not reflect the effects of alternative disinfection methods or repeated clinical disinfection cycles. The assessment was limited to compression resistance and tear strength, excluding other clinically relevant properties such as dimensional stability and detail reproduction. Additionally, the relatively small sample size and evaluation of limited material formulations may restrict the generalizability of the findings.

## Conclusions

This in vitro investigation demonstrated that chemical disinfection influences the mechanical performance of interocclusal recording materials to varying degrees. Polyvinyl siloxane maintained the highest levels of compression resistance and tear strength after immersion, indicating strong resistance to disinfectant-related mechanical alteration. Aluwax showed intermediate stability, whereas zinc oxide eugenol experienced the greatest reduction in mechanical properties following disinfection.

From a clinical standpoint, selecting materials that preserve their mechanical integrity after routine sterilization is essential for maintaining accurate interocclusal records and ensuring reliable prosthodontic outcomes. The findings emphasize that effective infection control procedures should be paired with materials capable of withstanding chemical exposure without significant compromise. Careful material selection can therefore support both clinical accuracy and patient safety.
